# Surgical Aspects of Intrahepatic Cholangiocarcinoma

**DOI:** 10.3390/cancers14246265

**Published:** 2022-12-19

**Authors:** Amram Kupietzky, Arie Ariche

**Affiliations:** Department of Surgery, Hadassah Medical Organization and Faculty of Medicine, Hebrew University of Jerusalem, Jerusalem 91905, Israel

**Keywords:** intrahepatic cholangiocarcinoma, biliary tract cancers, surgical resection

## Abstract

**Simple Summary:**

Intrahepatic cholangiocarcinoma (ICC) is a rare and aggressive malignancy, and its incidence is on the rise worldwide. Surgical resection is the only curative treatment of ICC. The various aspects of surgical management of ICC are discussed in this review.

**Abstract:**

Intrahepatic cholangiocarcinoma (ICC) is a rare and aggressive malignancy. It originates from the bile ducts and is the second most common primary cancer of the liver. Surgery is considered the only curative treatment of ICC, offering the best chance for long-term survival. The purpose of this article is to review the available literature on ICC, with a focus on the various aspects of the surgical care in this potentially lethal malignancy.

## 1. Introduction

Intrahepatic cholangiocarcinoma (ICC) is a rare and aggressive malignancy. It originates from the bile ducts and is the second most common primary cancer of the liver. ICC accounts for about 20% of liver and 3% of all malignancies [1,2]. Since the 1980s, the number of patients has been constantly rising worldwide: the highest numbers are reported in Southeast Asia, followed by Europe and the USA [3]. Although it had been demonstrated that ICC is mostly prevalent in elderly men, recent data suggests that the gender predilection is less dominant and that there is an uptrend of ICC in all group ages, even among patients younger than 45 years of age [3,4,5]. Biliary intraepithelial neoplasia and intraductal papillary neoplasm of the bile duct (IPNB) are the two main cholangiocarcinoma precursor lesions [6]. These lesions have an intraductal growth pattern consisting of a superficial spread of carcinoma cells along the biliary mucosa without invasive spread [7]. In addition to their premalignant pathophysiology, these lesions can cause symptoms (jaundice, pain, cholangitis etc.). IPNB accounts for 10–15% of bile duct tumors [8]. Complete surgical resection provides the best outcomes for these patients [9].

Risk factors associated with ICC are biliary cysts, primary sclerosing cholangitis (PSC), hepatolithiasis, cirrhosis, viral hepatitis, parasitic infections, and exposure to carcinogens. At least four known genetic conditions are associated with the development of ICC: Lynch syndrome, BRCA-associated protein-1 (BAP-1) tumor predisposition syndrome, cystic fibrosis, and biliary papillomatosis [10].

Global mortality rates are stagnant in most parts of the world [4,11,12,13,14]. While median survival has improved slightly over the last decade, five-year overall survival (OS) remains dismal at around 9% [2]. Complete surgical resection remains the only potential cure for ICC, but only one third of patients present with a tumor amenable to surgical resection. Even when patients undergo surgical resection with curative intent, five-year OS is 20–35% [15]. Patients with unresectable diseases who receive palliative care treatment have a median survival of 12.9 months [16].

Recurrence of ICC occurs in up to 70% of patients during the first five years after curative-intent resection, data that emphasize the aggressiveness of the disease [15]. The concentration of care to specialized centers with experienced multidisciplinary care teams has improved outcomes for patients with ICC [2,17,18]. The purpose of this article is to review the available literature on ICC, with a focus on the various aspects of the surgical care in this potentially lethal malignancy.

## 2. Preoperative Workup

Patients with cholangiocarcinoma classically present with newly onset jaundice, vague abdominal pain, itching, weight loss, acholic stools, and dark urine, though the extent of these biliary obstruction symptoms may vary based on the anatomic location of the tumor. Intra hepatic cholangiocarcinoma is less likely to cause obstructive symptoms, and symptoms are often indolent and non-specific accounting to delayed diagnosis in many cases [19]. Once a hepatic mass is suspected to be malignant, the workup should include blood workup for tumor markers (CEA, CA19-9 and AFP), imaging, including an abdominal CT (multiphase) or an MRI, and IV contrast and a chest CT for a metastatic workup. Tissue diagnosis consist of a histopathologic diagnosis via biopsy, though patients with a resectable disease do not necessarily need a biopsy before proceeding to surgery. Patients that can be waved from biopsy are those with typical imaging, elevated serum levels of CA19-9, and normal IgG4 levels [20]; these patients can proceed to upfront hepatic resection.

## 3. Imaging

The role of imaging in the work up of ICC is two-fold: it affirms the diagnosis, and it allows the evaluation of the extent of the disease, thus it allows us to assess the resectability of the primary mass and outlines the route of treatment. It is crucial to distinguish between HCC and ICC. As HCC is supplied mostly by the arterial hepatic blood supply, it tends to enhance during the arterial phase, while ICC receives most of its blood supply from the portal system, therefore it has a delayed phase enhancement pattern, hence the importance of a multiphase IV contrast CT [21]. MRI has similar advantages as the CT scan, yet it is better in detecting regional spread. Finally, it is often recommended to complete a FDG PET CT, as it is superior at detecting metastases compared to a CT scan or MRI, and can lead to a change in treatment in up to 30% of patients [22].

## 4. Staging

Before 2010, it was commonly accepted to stage ICC using the same TNM-based staging system as that of HCC. Since the America Joint Committee on Cancer (AJCC), in their seventh edition. published the first TNM staging system specifically for cholangiocarcinoma, it is now the most widely used staging system for ICC. In the eighth edition, they added additional prognostic factors, including a subgroup of T1 depending on the tumor size (with 5 CM as the cutoff), and downgraded lymph node metastasis from stage 4a to 3b (Table 1). It is also now recommended that the dissection should include at least 6 lymph nodes for an accurate N staging. However, recent studies suggest that the overall prognostic ability was not dramatically improved between the two editions [23,24,25,26].

## 5. Staging Laparoscopy

A diagnostic laparoscopy, as part of the perioperative evaluation, has been proposed as a staging modality to identify patients with occult peritoneal and hepatic metastases. An early detection of an unresectable disease can save patients from an unnecessary laparotomy. Early studies found that only 30–40% of patients undergoing on explorative laparotomy for ICC were found to a have a resectable disease [27,28]. In a recent relatively large retrospective study, of 80 patients with potentially resectable ICC, 35 patients underwent staging laparoscopy on the suspicion of distant metastases [29]. Fifteen patients were found to have an unresectable disease. However, when considering optimal use of preoperative imaging, staging laparoscopy precluded resection in seven patients. The authors conclude that to date, imaging has not completely replaced diagnostic laparoscopy in detecting unresectable ICC. The American Hepato–Pancreato–Biliary Association recommend a selective use of a diagnostic laparoscopy with intraoperative ultrasonography only in patients with high-risk features [30]. These include patients with a multicentric disease, high CA 19-9, a suspected vascular invasion, or peritoneal disease [30].

## 6. Indications for Surgery

Surgery is considered the only curative treatment of ICC, offering the best chance for long-term survival [31]. The aim of surgery in patients with ICC is to completely resect the tumor to achieve a R0 resection (negative microscopic margin) and perform a regional lymphadenectomy, while leaving a future liver remnant that is sufficient for the patient. Patients must be appropriate surgical candidates, able to withstand a major surgery and its potential comorbidities. At the time of diagnoses, only about 25% of patients are suitable for surgical resection [32]. Bilateral multifocal or multicentric disease is considered a metastatic disease and is associated in many studies with a significantly shorter overall survival and therefore is a relative contraindication to surgery [33]. Buettner et al. investigated long term survival in 1013 patients who underwent resection of ICC and found that the median survival of patients with a solitary ICC was 43.2 months vs. 21.2 months with 2 tumors, vs. 15.3 months in patients with 3 tumors or more [34]. While major vascular resection was considered somewhat a relative contraindication for surgery, it is now considered feasible and can be performed safely in experienced centers with an acceptable median overall survival of 33.4 months [7]. Up to date, four major consensus guidelines were published on the indications for resection of intrahepatic cholangiocarcinoma: the International Liver Cancer Association guidelines in 2014, the Americas Hepatopancreatobiliary Association Guidelines in 2015, the National Comprehensive Cancer Network Guidelines in 2019, and the European Network for the Study of Cholangiocarcinoma Expert Consensus Statement of 2020. All guidelines seem to agree that patients with a extrahepatic disease, distant lymph node metastases, or intrahepatic metastatic disease (multiple bi-lobar or multicentric tumors), will not benefit from surgical resection and are contraindicated to surgery [20,30,35,36].

## 7. Surgery

### 7.1. Tumor Resection and Margins

The only treatment for ICC associated with overall and disease-free survival is surgical resection with negative margins. Spolverato et al. found that one of six patients undergoing a hepatic resection due to ICC will have a positive margin, which was associated with a poor OS [37]. The impact of surgical margin width on long-term survival has been up to debate and was recently investigated by Liu et al. in a multicenter study [38]. They found that a surgical margin of 10 mm or more was associated with a longer OS of 41 months compared to 22 months in patients with a narrow surgical margin of less than 10 mm. When preforming a subgroup analysis, this was true only in patients with AJCC stage I ICC. The OS of patients with AJCC stage II or III was not affected by the surgical margin and was around 15 months regardless to the surgical margins.

While previous studies supported anatomic resections for HCC, recent data suggests that non-anatomic hepatectomy is equivalent to anatomic resections for ICC [39,40,41]. Zhang et al. in a recent study compared anatomical vs. non anatomic resection of ICC and found that anatomical major hepatectomy for ICC did not improve the overall survival and was associated with increased perioperative morbidity, they conclude that the margin width, rather than the extent of resection, affects long-term outcomes [42].

### 7.2. Future Liver Remnant

When evaluating a patient with ICC for surgical resection, it is not enough to reach a clean margin resection, but the patient must also be left with an adequate future liver remnant (FLR). The FLR is commonly composed of two continued liver segments with their adequate vascular perfusion, venous outflow, and biliary drainage reaching a volume that is adequate for the patient [43]. The exact volume remanent that is considered an adequate one depends on the functional state of the remaining liver; a 20% remanent of the total liver volume should suffice with a normal functioning liver [44]. Patients with chemotherapy associated liver injuries or fatty livers will need about a 30% remanent, and patients with fibrosis or cirrhosis will need a remnant that is at least 40% of their original liver volume [44]. The calculation of the FLR is based on cross-sectional imaging, with either CT scans or MRI scans [45,46]. Even when surgery seems to be impossible due to insufficient FLR, liver augmentation can be considered. Based on the regeneration ability of hepatic tissue, by occluding the portal vascular supply to the tumor bearing are of the liver that is planned for resection, the remining liver tissue should hypertrophy [47]. Several techniques have been described including portal vein embolization (PVE), liver venous deprivation (LVD), and the recently described techniques associating liver partition and portal vein ligation (ALPPS), and the combined PVE/hepatic vein embolization (HVE) [48,49]. These strategies have been shown to accelerate liver regeneration and achieve sufficient FLR in patients that where otherwise considered poor surgical candidates [33,50].

### 7.3. Transplantation

Historically, liver transplant was contraindicated for patients with ICC due to poor outcomes and early disease recurrence [51]. Several recent studies have found that patients who had undergone transplants for what was suspected as HCC yet on pathology was ICC had up to a 73% survival rate at 5 years [52,53]. A recent review published by Sun et al. concluded that patients with ICC that can benefit from liver transplant are those with very early-stage disease or those with advanced-stage ICC that responded well to neoadjuvant chemotherapy [54]. It seems that future research is warranted to establish whether liver transplant should be part of our standard of care for ICC and establish a universal criterion for patient selection eligible for this treatment [55].

### 7.4. Lymphadenectomy

**Figure 1:**
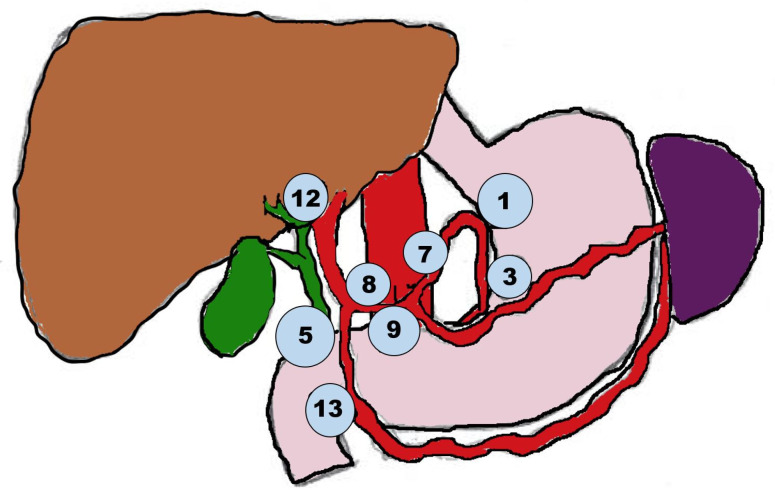
Lymph node locations for ICC lymphadenectomy.

Lymph node dissection (LND) has been an integral part of surgical treatment for ICC and current guidelines recommend that at least six nodes should be harvested during surgery [24]. It is recommended that the dissection should include at least stations no. 12 (within the hepatoduodenal ligament) and 8 (along the common hepatic artery) for accurate staging, regardless of tumor location (Figure 1) [56,57]. Kim et al. found that the number and location of positive nodes plays an important role in staging and assessing prognosis accurately, though the role of lymph node dissection on locoregional control remains questionable [58]. Hu et al., in their recently published multicenter retrospective study, found that despite the above guidelines only 76% of patients who underwent a radical surgery for ICC underwent LND, and just 37% of patients had a minimum of 6 lymph nodes dissected, though when comparing prognosis between patients who had LND and those who had not, they found no statistical significance in prognosis between the two [58]. A recent study investigated the use of hepatic arterial infusion chemotherapy with floxuridine as an alternative option for locoregional treatment for IHC and found that it was comparable to resection, and both options were better than systemic chemotherapy alone regarding overall survival [59]. A new nodal staging has recently been proposed with N1 including 1–2 positive lymph nodes, and N2 including ≥3 positive lymph nodes, to stratify more precisely prognosis, as new accumulating data suggests that patients with 3 or more lymph node metastasis may have a worse prognosis than patients with 1–2 positive nodes [57].

### 7.5. Minimally Invasive Surgery

Laparoscopic liver resection for malignant liver diseases has been gaining popularity in the past several years, offering the benefit of minimal invasiveness, including shorter hospital stay length and less complications compared to an open surgery. At this point, there is no randomized comparative study that compared open vs. minimally invasive surgery (MIS) for ICC. A recent systematic review by Patrone et al. found 9 comparative retrospective studies on MIS for ICC and included 3012 patients, concluding that MIS is feasible and safe for ICC in patients with a tumor diameter <5 cm, without main biliary duct invasion, without large vascular invasion, and in which biliary and vascular reconstructions were not needed [60]. A lower intraoperative blood loss and significantly decreased postoperative hospital stay were also found in the MIS patients. These advantages for MIS were was also found in a recent multicenter data-based matched study by Jinhuan et al. [61]. Though the immediate and short-term advantages of MIS for ICC in selected patients seem to be agreed upon, further research is warranted regarding the long-term oncologic outcomes and the impact on patients’ quality of life.

## 8. Recurrence

Even after a successful resection and adjuvant systemic chemotherapy, the recurrence of ICC is extremely high, with recurrence rates of 40–76% within 2 years after surgery, attributing to a 5-year survival rate of 25–43% [62,63,64,65]. Recurrence within the first two years after resection is commonly defined as early recurrence, and is associated with a worse prognosis [15]. A recent retrospective study of 933 patients with ICC with a median follow up of 22 months found that, of 685 patients with a recurrence after a curative resection, the overall survival from the time of recurrence was worse among patients who had early versus late recurrence (median 10 versus 18 months, respectively; *p* = 0.029) [15]. Recurrence can occur in the resection margin, other intrahepatic sites, or extrahepatically (including lungs, peritoneum, lymph nodes, bone, and adrenal glands), with most recurrences occurring in the liver [19,64,66]. There is increasing evidence that aggressive treatment including re-resection may prolong survival [67,68]. A recent multi-institutional study by the Okayama Study Group of HBP surgery, consisting of 345 cases of ICC with 223 recurrences, found that surgical resection of recurrent masses showed a median survival time of 39.5 months, significantly better than those treated non-surgically [69]. They conclude that resection can provide clear survival benefits to patients with intrahepatic-only or extrahepatic-only recurrence, however they did not find any benefit in surgical treatment when simultaneous intra and extrahepatic recurrence occurred.

## 9. Neoadjuvant and Adjuvant Therapy

The BILCAP trial, published in 2019, showed on a per-protocol analyses that adjuvant therapy with Capecitabine improved OS compared to observation following radical surgery for biliary cancer by almost one third, though in the intention-to-treat analysis, this benefit was not significant [70]. Of the 447 patients included in this trial, only 84 had ICC. Controversially, the PRODIGE 12 study, a phase III randomized clinical trial that included 196 patients, who were operated upon due to biliary cancers, failed to prove any benefit with adjuvant gemcitabine and oxaliplatin chemotherapy compared to surveillance only [71]. This trial included 89 patients with ICC. Based on up-to-date clinical knowledge, it is difficult to reach a practical consensus regrading adjuvant chemotherapy for ICC, and while additional adjuvant clinical trials are currently accruing, we shall have to wait and see what the results of these may suggest in the future [72]. Regarding neoadjuvant chemotherapy, the evidence is even less persuasive, as there have been no prospective randomized trials assessing the value of neoadjuvant chemotherapy with ICC. A few retrospective studies, consisting of 1880 patients in total, have shown that neoadjuvant chemotherapy can down-stage unresectable ICC, and increase the proportion of patients eligible for surgery (38%, of them 60% R0 resections), in turn improving the outcomes of patients with ICC [73].

## 10. Targeted and Immune-Based Therapy

Several genetic abnormalities have been identified in ICC, including alterations such as TP53, ARID1A, and KRAS, though specifically FGFR and IDH seem to be quite promising in the pursuit of specific therapeutic targets for ICC [74,75,76]. Promising trial reports regarding the use of FGFR inhibitors such as Pemigatinib, Futibatinib, and Infigratinib have supported the use of FGFR inhibitors in the treatment of ICC [66,77,78,79]. As targeted therapies emerge, patients with advanced ICC should have tumor genetic profiling preformed to appropriately enroll in ongoing clinical trials.

## 11. Conclusions

ICC is a rare, potentially lethal malignancy that usually presents at advanced stages due to extended periods of vague symptoms. Accurate staging and planning of the therapeutic options including state-of-the-art imaging in combination with laboratory work-up are needed. Hepatic resection with negative surgical margins and a lymphadenectomy with evaluation of ≥6 nodes should be the standard of care. Adjuvant chemotherapy with capecitabine is highly recommended following surgical resection of ICC, given the high incidence of post-operative recurrence. More studies should be performed to establish the role of alternative treatment options, including transplantation, as well as targeted therapy in the treatment of patients with ICC.

## Figures and Tables

**Table 1:** The AJCC 8th edition Staging System for ICC.

T1	Solitary Tumor without Vascular Invasion
T1a	Solitary tumor ≤ 5 cm without vascular invasion
T1b	Solitary tumor > 5 cm without vascular invasion
T2	Solitary tumor with intrahepatic vascular invasion or multipletumors, with or without vascular invasion
T3	Tumor perforating the visceral peritoneum
T4	Tumor involving local extrahepatic structures by direct invasion
N0	No regional lymph node metastasis
N1	Regional lymph node metastasis present
M1	Distant metastasis
IA	T1aN0M0
IB	T1bN0M0
II	T2N0M0
IIIA	T3N0M0
IIIB	T4N0M0, TAnyN1M0
IV	TAnyNAnyM1
